# Highlighting rare disease research with a *GENETICS* and *G3* series on genetic models of rare diseases

**DOI:** 10.1093/genetics/iyad121

**Published:** 2023-08-09

**Authors:** Philip Hieter, Brenda Andrews, Douglas Fowler, Hugo Bellen

**Affiliations:** Michael Smith Laboratories, Department of Medical Genetics, University of British Columbia, Vancouver, BC, Canada; The Donnelly Centre and Department of Molecular Genetics, University of Toronto, Toronto, ON M5S 3E1, Canada; Department of Genome Sciences and Department of Bioengineering, University of Washington, Seattle, WA, USA; Departments of Molecular and Human Genetics, and Neuroscience Neurological Research Institute, Texas Children's Hospital Baylor College of Medicine, Houston, TX, USA

To highlight the development of new model systems, assays, and resources designed to illuminate rare disease gene function, the Genetics Society of America has launched a special collection of publications in the GSA journals *GENETICS* and *G3*: *Genes|Genomes|Genetics*. This editorial accompanies 9 papers that describe the development of genetic approaches to study rare disease in 4 well-established model systems—yeast (*Saccharomyces cerevisiae*), worm (*Caenorhabditis elegans*), fly (*Drosophila*), and mouse. In most cases, the assays exploit orthologues of rare disease genes in the model organism of choice and use genome engineering to introduce rare disease variants for subsequent phenotypic analysis.

The need for model organism-based studies to provide functional insights into human disease has never been greater. Over the past 10 years, a staggering ∼4–5 new rare disease-gene associations have been discovered per week. After the first few rare disease gene discoveries by whole-exome or whole-genome sequencing in early 2010, the rate dramatically accelerated to approximately 250 rare disease genes per year in 2012. This rapid increase in the rate of discovery was driven by the four orders-of-magnitude reduction in the cost of DNA sequencing in the late 2000s through the development of next-generation sequencing methods. As a result, the comprehensive sequencing of small groups of patients exhibiting the same phenotype and mining successfully for the disease gene carrying the causal genetic variants became both feasible and affordable. Currently, the Online Mendelian Inheritance in Man (OMIM) documents 4,434 disease genes that, when mutated, are the causal basis of 6,306 Mendelian disorders (https://www.omim.org/statistics/geneMap). Two hundred and sixty-three million individuals globally are estimated to have a rare disease, and one estimate predicts an additional 6,000–13,000 Mendelian conditions for which the disease-gene mutations remain to be discovered (Bamshad *et al*. 2019). Therefore, we can expect the causal mutations for thousands of additional “unsolved” rare diseases to be discovered in the next decade.

The initial discovery of a rare disease gene and the genetic variants that cause the disease phenotype represents a monumental breakthrough for patients, their families, and clinicians. The discovery provides a definitive DNA-based diagnostic for patients and links patients, clinicians, and advocates in a common cause to find cures. Model organisms are now playing a growing role in the diagnosis of individuals with rare diseases (Wangler *et al* 2017). When only one or very few individuals are affected with rare, potentially damaging variants in the same gene, genetic approaches in model organisms can contribute substantially to the diagnosis. Most importantly, following the discovery of the human variants, the question becomes how to use this information to the benefit of patients. The answer is almost always to be found in functional studies that reveal the biological consequences of the variants and a detailed mechanistic understanding of the genes and pathways involved. Much can be learned about mechanistic aspects of human diseases through analysis of orthologous genes and pathways in experimentally tractable organisms using the suite of experimental tools developed for each model organism (yeast, fly, worm, zebrafish, mouse, and others). These model organisms have complementary biology and experimental tools; therefore, functional analysis in many organisms is more powerful than in any single organism.

In addition to revealing how disease-associated variants act, model organisms can be used to develop therapeutics. For example, screening drugs in yeast, worms, and flies has yielded numerous successes and is more affordable than screening vertebrates. Hits can be validated or refuted in vertebrate model organisms such as fish and mice. Thus, model organism studies deliver significant benefits to patients through advancements in knowledge of disease and human biology leading to potential strategies for the prevention and/or treatment of rare diseases.

Four papers from the series use genome engineering or transgenic approaches to introduce disease causing genetic variants into the corresponding ortholog of the model organism, to gain insights into the biological impacts of specific disease-associated variants, better understand disease pathogenesis, and/or establish a model for therapeutic discovery. Gruss *et al* (2023) used CRISPR/Cas9 genome editing to create a variety of mutations in the predicted TWIST box of helix loop helix (HLH)-8, which is the worm counterpart of human TWIST1, a transcriptional regulator. Autosomal dominant pathogenic variants in *TWIST1* lead to craniofacial defects in Saethre–Chotzen syndrome (SCS), and these patient variants and various alanine substitutions were engineered in the worm gene. Straightforward phenotypic assays enabled the team to discover that patient mutations were not equivalent to alanine substitution mutations, which are complete loss-of-function, suggesting that patient variants may be only partial-loss-of-function alleles. Future work involving assessment of interacting proteins or mutations in the genetically accessible worm model promises to shed light on the defects in SCS patients. Sterrett *et al* (2023) used a multipronged approach to explore the phenotypes associated with introducing disease-causing variants into the yeast gene *rrp4*, which is an ortholog of the human *EXOSC2* gene. These genes encode the cap subunit of the RNA exosome, and a missense mutation was identified as a rare patient mutation in multiple myeloma, in a residue implicated in mediating a critical interaction with the MTR4 RNA helicase. The phenotypes associated with the analogous mutation in yeast (rrp4-M68T) were assessed using tests for genetic and chemical–genetic interactions, expression of RNA exosome targets, and effects on functionally relevant protein–protein interactions. This complementary suite of assays revealed that the patient mutation affects the interaction between Rrp4 and the Mtr4, likely impairing exosome function toward a subset of Mtr4-dependent targets. This study beautifully illustrates the power of accessible model systems to provide significant mechanistic insights that can impact our understanding of rare diseases pathology. Jangam *et al* (2023) illustrate the synergy between model organism and human genetics by developing a fly model to investigate a rare missense variant in *EZH1* in a patient with neurodevelopmental delay and hypotonia. *EZH1* encodes a component of the Polycomb repressive complex and this is important for regulating both chromatin state and gene expression. Previously, *EZH1* had not been linked to any Mendelian disease. Because the fly gene, *Enhancer of zeste E(z)*, is orthologous to human *EZH1*, the authors produced a fly model of the human variant by making the analogous mutation, *E(z)^A691G^.* When overexpressed, *E(z)^A691G^* altered histone marks and caused a dramatic homeotic patterning defect relative to *E(z)^WT^*, suggesting that the variant can act in a dominant gain-of-function manner. Bergwell *et al* (2023) used CRISPR/Cas9 genome editing to develop a *C. elegans* model of autosomal recessive primary microcephaly through introduction of a mutation within the highly conserved core centriole gene, *sas-6*. *C. elegans* carrying the pathogenic variant exhibited shortened phasmid cilia, abnormal cilia morphology, shorter dendrites, and chemotaxis defects. The development of this *sas-6*(L69T) *C. elegans* model will be useful to study the effects of additional disease-associated human variants in the future.

One paper uses an alternative approach that involves ectopic heterologous expression of genes involved in human disease in a model organism. This enables phenotypic analyses of disease genes and variants that may not have an obvious ortholog in the model organism genome. Swanson *et al* (2023) report the novel finding that the telomere-binding protein Rap1 plays an extratelomeric role in the cytoplasm through its interactions with a cell type-specific isoform of glial fibrillary acidic protein (GFAPε) and presenillin 1 (which processes amyloid precursor protein and is associated with Alzheimer's disease). Using a humanized yeast reconstitution system of γ-secretase activity, they showed that RAP1 increased γ-secretase activity, and the effect was enhanced by GFAPε. These discoveries reveal previously unknown mechanisms that may suggest novel targets for Alzheimer's disease therapies.

Another paper describes the creation of a mouse model of a congenital disease caused by a gain-of-function embryonic lethal mutation that occurs in somatic cells and allows survival by mosaicism. Sturge–Weber syndrome is caused not by germline mutations but by a somatic mutation in the *GNAQ* gene. It was argued decades ago by Happle (1987) that this condition is an example of a “lethal mutation that survives by mosaicism.” Wetzel-Strong *et al*. (2023) created a mouse model in which they conditionally expressed the p.R183Q mutant from the endogenous *Gnaq* locus. The authors found that some mosaic animals died during embryogenesis whereas other survived. Those that were phenotyped in embryogenesis had edema and dilated small vessels, in agreement with what was observed in mosaic tissue in patients. In contrast, mice in which the mutation was induced in all tissues died in embryogenesis with vasodilation and hemorrhage arguing that it is indeed a dominant lethal mutation that allows survival into adults only in mosaic animals. Their data support that this allele of *Gaqm* is indeed a “lethal mutation that survives by mosaicism” in human, providing compelling in vivo evidence that support Happle's (1987) hypothesis.

Cao *et al* (2023) studied the interactions between 2 genes in mice that affect phosphoinositides. Complete or strong loss of FIG4 (encoding polyphosphoinositide phosphatase) causes a lethal multisystem disorder Yunis–Varon syndrome with severe bone and neurologic features, whereas partial loss of FIG4 causes a peripheral neuropathy: Charcot-Marie-Tooth Type 4J. Interestingly, inhibition of a phosphoinositol 4 kinase, PIP4K2C, in cultured cells increased the level of phospholipid [PI(3,5)P2], while partial loss of PIP4K2C elevates PI(3,5)P2, resulting in enhanced autophagy and increased turnover of mutant Huntington protein (Al-Ramahi *et al*. 2017). In contrast to mice that lack *Fig 4*, mice that lack the *Pip4k2c* gene are viable and healthy. Cao *et al* (2023) evaluated the interaction between *Fig 4* and *Pip4k2C* in mice; the authors created *Fig 4−/−; Pip4k2c−/+* mice. This led to a modest but increased survival of the double-mutant animals and improved lysosomal morphology. Their data suggest that small molecules inhibitors of PIP4K2C may have therapeutic potential and should be further explored in animal models.

The genetic causes of intellectual disability (ID) have been a rapidly expanding list of genes and genetic pathways. An important subgroup of these genes is associated with the SWItch/Sucrose Non-Fermentable (SWI-SNF) complex and is called SSRIDDs for “switch/sucrose nonfermentable related intellectual disability disorders.” Another group of ID genes cause Cornelia de Lange Syndrome (CdLS), a disorder of chromatin modification associated with the cohesin complex. MacPherson *et al*. (2023) systematically explore the consequences of a reduction of the expression of 3 genes associated with CdLS and 3 genes associated with SRIDDs using a set of assays including a startle-induced locomotor response, sleep and activity, brain size, as well as RNA sequencing followed by a detailed analysis of differentially expressed genes in flies. Interestingly, the flies with ubiquitous RNAi knockdown showed an increase in overall spontaneous activity, a decrease in night sleep and a fragmented sleep. Brain morphological studies showed variable phenotypes, and only knockdown of fly *osa* (orthologous to human *ARID1A* and *ARID1B*) showed consistent strong phenotypes. Clustering analysis revealed that genes are coregulated within and across SSRID and CdLS models. The data gathered from these studies provide the basis to further probe molecular effects of variants in chromatin modification pathways.

Finally, Kaldunski *et al* (2023) provide a useful overview of the resources available for rare disease research at one of the model organism genome databases. They highlight the Rat Genome Database with consolidated data about rare diseases, associated genes, and relevant rat strains/cell lines, as well as links to a variety of useful analysis tools. This paper highlights the central role that model organism databases have played in nurturing productive communities of model organism genetics and continue to play in advancing our understanding of human biology. In this regard, it is important to recognize the efforts of the Alliance of Genome Resources to integrate data across 7 model organism knowledgebase projects (Saccharomyces Genome Database, WormBase, FlyBase, Mouse Genome Database, the Zebrafish Information Network, Rat Genome Database, and the Gene Ontology Resource) and increase accessibility by the basic science and clinical research communities (Alliance of Genome Resources Consortium 2022).

We anticipate that many more studies will focus on modeling the genetic variants observed in individuals with rare diseases, aiding in diagnosis of undiagnosed rare disease patients, providing an understanding of the nature of the pathogenic variants (loss- vs gain-of-function), revealing conserved biological pathways that are affected, providing insights into molecular and cellular mechanisms, and guiding development of therapeutic approaches. An important aspect of this work will be communication and collaboration between clinicians discovering new disease genes in rare disease patients and basic scientists who can analyze equivalent genes in model organisms. We therefore hope to receive more papers describing model systems that address these important goals and to keep the community abreast of new developments and resources in established model organism and developing model system databases.
